# Challenges in the delivery of public HIV testing and counselling (HTC) in Douala, Cameroon: providers perspectives and implications on quality of HTC services

**DOI:** 10.1186/s12914-017-0118-2

**Published:** 2017-04-08

**Authors:** Patrice Ngangue, Marie-Pierre Gagnon, Emmanuelle Bedard

**Affiliations:** 1 Faculty of Nursing Sciences, Laval University, 1050, avenue de la medicine, Pavillon Vandry, Québec, Québec G1V0A6 Canada; 2 Population Health and Optimal Health Practices, CHU de Québec Research Center, 10, Rue de l’Espinay, D6, Québec, Québec G1L 3L5 Canada; 3 Université du Québec à Rimouski (UQAR), 1595 Boul. Alphonse-Desjardins, UQAR, Campus de Lévis, Lévis, Québec Canada

**Keywords:** Quality of health services, HIV testing and counselling, Cameroon, Lay counsellors, Qualitative study

## Abstract

**Background:**

The Cameroon government has made HIV testing and counselling (HTC) a priority in its HIV/AIDS strategic plan. However, there is a dearth of literature on the perspectives of providers on the quality of HTC services. The aim of this study was to explore challenges in the provision of HTC services and their implications on quality of HTC services in Douala’s district hospitals.

**Methods:**

Two primary data collection methods supported by the Donabedian’s model of healthcare were used to explain the challenges in the provision of HTC services and their implications on quality of HTC services. This consisted of semi-structured individual interviews with 6 nurses and 16 lay counsellors and a non-participant observation of the physical environment for HTC by site. The study sites were the prevention and voluntary testing and counselling centre (PVTCC) of the six district hospitals of the city of Douala.

**Results:**

The study reveals concerns about confidentiality and privacy during the counselling sessions due to inadequate and limited space. An absence of consent, even verbal, was reported in one PVTCC. There is no specific accredited training curriculum that leads to a formal registration as a PVTCC staff, and some lay counsellors work without training. Lay counsellors carry the burden of HIV counselling, but the majority of them work for many years without remuneration and recognition. Another quality challenge is the high workload in the district hospitals’ lab, which leads to long waiting times for HIV test results, thus contributing to failure to return for results.

**Conclusion:**

The findings of this study highlighted some issues such as lack of adequate space and equipment for HIV testing and counselling that hinder the quality of HTC services and should challenge the health authorities of Cameroon on the need to reorganize HTC services and create a national HIV quality assurance program.

**Electronic supplementary material:**

The online version of this article (doi:10.1186/s12914-017-0118-2) contains supplementary material, which is available to authorized users.

## Background

Despite the introduction of other HIV testing and counselling (HTC) modalities such as community-based HTC, many people in sub-Saharan Africa continue to be tested in health facilities [1, 2]. For that reason, these testing and counselling structures should ensure high quality services [3]. Good quality HTC can contribute to the early detection of HIV infection among the population, which can facilitate early access to interventions and support services [2]. Additionally, through education provided during pre and post-test counselling, prevention messages can be promoted in a targeted manner [2, 3]. Nevertheless, a regular and continuous quality assessment of the counselling and screening activities has to be conducted in each country burdened by HIV, especially in Sub-Sahara African countries [2–4]. However, in these countries, one of the biggest issues faced by the health system is the shortage of human resources of health. To address this problem, in the era of HIV testing counselling, many African countries such as Cameroon, introduced task shifting [5]. In this perspective, HIV testing and counselling are under the responsibility of lay counsellors and nurses. However, this expansion might affect quality of HTC services in different ways [6]. For example, in many studies conducted in sub-Saharan Africa, providers reported lack of confidentiality and privacy due to limited space, difficulties in upholding consent in provider-initiated testing, high workload, inadequate training and supervision as key elements impacting on quality of counselling and testing [7–10].

In Cameroon, data from the 2011 Demographic and Health Survey (DHS 2011) show that 4.3% of people between 15 and 49 years of age are HIV-positive. The HIV prevalence among women aged 15–49 (5.6%) is almost twice as high as for males of the same age bracket (2.9%) [11]. The Cameroon government’s response to HIV infection includes several strategies such as strengthening the prevention of transmission of HIV and other sexually transmitted infections (STIs) and access to care and treatment. Given that the knowledge of one’s HIV status is important for reinforcing prevention strategies and an early therapeutic intervention, the Cameroon government has made HTC a priority in its strategic plan. The ambitious 2011–2013 operational plan which aimed at screening 1,236,803 people resulted in the screening of about 512,087 (41%) people who effectively received counselling and underwent voluntary testing [12]. In addition, it is estimated that a significant proportion of infected women (30%) and infected men (36%) had never tested for HIV or had undergone testing but ignored the results [11]. In a recent retrospective analysis of HTC records of the six district hospitals of Douala, study (January, 1^st^ 2009 to December, 31^st^ 2013), we found an overall failure to return (FTR) rate for HIV testing and counselling of 14.3% [13].

In public health facilities such as district hospitals, HTC services are part of the HIV district management units (DMU). Besides the counselling and testing activities, DMU are responsible for the initiation of antiretroviral treatment, therapeutic education of new and old patients on antiretroviral treatment, psychosocial support and possible complications and side effects that might occur [14]. The DMU are usually managed by a head-nurse under the supervision of a doctor whereas non-health care providers (lay counsellors) ensured the counselling. To our knowledge, in Cameroon, there is no study focused on the quality of HTC services and HTC staff experiences and opinions. Therefore, the aim of this study was to explore challenges in the provision of HTC services and their implications on quality in Douala’s district hospitals.

## Methods

To explain the factors associated to the quality of HTC services, we used the “structure-process-outcome” model of healthcare described by Donabedian [15, 16]. According to Donabedian, structure (e.g., attributes of material, human resources and organizational structure) influences process (what is actually done in giving and receiving care), which in its turn influences outcomes (health status). “Structure” can be thought of as the physical setting in which care takes place, the organization of care and the qualifications of providers. “Process” denotes the good practice for better performance of the care system (technical and interpersonal), the adequacy between care and the best professional practice. “Outcome” measures the impact of care on the health status of patients [15–18]. We chose Donabedian’s model as it is widely used and allows policymakers to conceptualize the underlying mechanisms that may contribute to the quality of HTC services [19–21]. In this study we focused on the HTC physical structure (location and material) and the organizational structure (human resources), the testing and counselling procedures (client welcoming, pre-test counselling, the blood sampling, the post-test counselling, waiting time for test results).

### Study design, setting, sampling and participants

This qualitative study consisted of the prevention and voluntary testing and counselling centre (PVTCC) of the six district hospitals (DH) of the city of Douala. These health centers were chosen primarily because they are publicly funded health facilities in which national health policies are implemented, and secondly because of their strategic geographical locations which allow to reach a large part of the population of Douala. These facilities provided the bulk of HTC services in Douala.

Douala is the capital of Cameroon’s Littoral Region and the country’s economic capital. The city is the most populated urban centre in Cameroon [22], with about 1,907,479 habitants. According to the 2011 Cameroon Demographic and Health Survey (DHS 2011), the prevalence of HIV/AIDS reached 4.6% for people aged 15 to 49 years in the city of Douala. Women were particularly affected, with a prevalence of 6.4% as opposed to 2.6% for men [11].

The study participants consisted of lay counsellors and head nurses working in the selected PVTCCs. Purposive sampling were used to select 16 lay counsellors and six nurses. This sampling technique was chosen to enable different experiences to be obtained from the participants within and across the sites.

### Data collection

This study used two primary data collection methods during five months (November 2013 to April 2014): semi-structured interviews with HTC service staff (nurses and lay counsellors) and a non-participant observation of the physical environment such as counsellors’ offices, waiting rooms, HIV testing space and equipment. Using observations in addition of semi-structured interviews may allow to have a better understanding of the phenomena under study and increase the validity of the study [23].

### Semi-structured interviews

The semi-structured interview guide was developed based on Donabedian’s model of quality of care [15, 16]. The interviews were conducted in French by the principal investigator (PI) and were audio-recorded. The interviews are conducted in the lay counsellors’ offices. A short introduction prior to the interview was used to explain the research goals and to make participants feel at ease. At the beginning of each interview, the participants were invited to introduce themselves (age, education level, job experience in the current service, and job description). Then, the interviewer asked participants their opinion on the testing and counselling process, and the organizational structure such as the availability and adequacy of human resources, material, funding, staff training, the use of rapid tests, results promptness. Finally, to evaluate the outcome of quality, participants were asked to discuss about the link between the organizational structure, the testing and counselling process and the return for test results and post-test counselling (Additional file 1).

### Observations

Observations of the PVTCCs’ physical environment were made during the PI visit and took place in the afternoon. In order to determine the type and nature of the information to be collected, an observation guide in the form of a check-list was developed based on the World Health Organization’s field-test version handbook for improving HIV testing and counselling services [3]. Data concerned the description of infrastructures and equipment, supplies and storage, and the conduct of the workday. The information collected was recorded in the logbook.

### Data analysis

Field notes and interviews were transcribed verbatim in French. Transcripts were entered into QDA Miner of Provalis research (version 4.1.19) qualitative software [24]. Direct content analysis was the selected method [25, 26]. The Donabedian’s theoretical framework [16] was used to organize the initial code-frame. The code-frame was refined after applying it to the first few transcripts, adding sub-dividing and combining codes. Both inductive and deductive approaches were used for coding. Coding was done inductively to capture new concepts and deductively for predetermined categories. The principal investigator read several times and coded all transcripts. A sub-sample of transcripts was double-coded (by PN and MPG) using the same initial code-frame. Each researcher’s revised code-frame was then compared and discussed to ensure all emerging concepts were captured. Codes with similar meanings were grouped under the same category. After the initial analysis, the grouped categories were refined. All analyses were performed in French and subsequently translated into English by the primary author. The results were discussed amongst the research group members. Discrepancies in opinions were discussed, and consensus was reached on all themes and codes text segments. This article adheres to the (Consolidated criteria for reporting qualitative research (COREQ) guidelines.

## Results

This study generated data on the quality of care aspects (structure, the process and outcomes) of HIV testing and counselling services in the district hospitals of the city of Douala. The results are presented according to this order. In addition, the profiles of the PVTCCs based on field observations are presented in Table 1.

**Table 1:** Profile of studied HIV testing and counselling centers of Douala, Cameroon

	PVTCC1	PVTCC2	PVTCC3	PVTCC4	PVTCC5	PVTCC6
1. Infrastructure						
• Facility registered to provide VCT services?	Yes	Yes	Yes	Yes	Yes	Yes
• Adequate counselling room/s available (well lit, spacious, ventilated, private, equipped with 3 chairs and 1 table)?	No	No	No	No	No	Yes
• Room (s) is equipped to accordingly perform a test?	No	Yes	No	No	No	No
• Penile model available and on display?	No	No	No	No	No	No
• Room/s and waiting area well maintained and clean?	Yes	Yes	No	No	Yes	Yes
• Adequate waiting area (chairs and space)?	Yes	No	No	No	Yes	Yes
2. Supply and storage						
• Uninterrupted and adequate supply of rapid test kits in stock?	No	No	No	No	No	No
3. Referral system						
• Referral system in place and functioning?	Yes	Yes	Yes	Yes	Yes	Yes
• Referral directory/list available?	Yes	Yes	Yes	Yes	Yes	Yes
• Designated referral site for care and support?	Yes	Yes	Yes	Yes	Yes	Yes
• Post-test support available (post-test counselling, people living with HIV, etc.)?	Yes	Yes	Yes	Yes	Yes	Yes
4. Record and information system						
• Uninterrupted and adequate supply of VCT data forms and client cards?	No	No	No	No	No	Yes
• System for anonymous client coding in place and functioning?	No	No	Yes	No	No	No
• Easily retrievable copies of quarterly reports sent to the regional level available?	Yes	Yes	Yes	Yes	Yes	Yes
• Accident/incident book available and used??	Yes	Yes	Yes	Yes	Yes	Yes
5. Information, Education & Communication (IEC) materials						
• Signboards, signs, labels and directions for VCT room/s?	No	No	No	No	No	Yes
• Opening hours prominently displayed?	No	No	No	Yes	No	Yes
• Door tags used for privacy (please enter/counselling in progress)?	No	No	No	No	No	No
6. Financial management						
• Fee charges prominently displayed?	No	No	Yes	No	No	No
• Records of accounts available?	Yes	Yes	Yes	Yes	Yes	Yes
• Clear policy and measures in place for clients unable to pay?	No	No	No	Yes	No	No
7. Guidelines/client-provider interaction						
• Did VCT staff in charge of counselling attended an official training programme and obtained a degree?	Yes	No	No	Yes	Yes	Yes
• Did VCT staff performing the rapid test attended an official training programme and obtained a degree?	No	No	No	No	No	No
• VCT services available on advertised days?	Yes	Yes	Yes	Yes	Yes	Yes
• Informed consent (signature) obtained before HIV testing?	No	No	No	No	No	No
• Condoms supplied where appropriate?	No	No	No	Yes	No	Yes
• Same-day blood testing conducted on site?	No	No	No	No	No	No
• Correct testing algorithm used?	Yes	Yes	Yes	Yes	Yes	Yes
• Test results given same day?	No	No	No	No	No	No
• All forms are checked for missing items at the end of each day?	No	No	No	No	No	No
• Community mobilization activities being conducted?	No	Yes	No	Yes	No	Yes
• All counsellors attending regular group supervision?	Yes	Yes	Yes	Yes	No	Yes
• All counsellors receiving individual or peer supervision?	Yes	Yes	Yes	Yes	Yes	Yes
• Counsellors working scheduled hours (not assigned to other non-VCT services)?	Yes	Yes	Yes	Yes	Yes	Yes
• Each counsellor sees <10 clients/day?	Yes	Yes	Yes	Yes	Yes	Yes

### Demographic characteristics of the research participants

The providers were lay counsellor (*n* = 16) and nurses (*n* = 6). Four head nurses of the HTCC and two assistant-nurses participated in the interviews. All were female with a mean age of 42.2 (26–60). On average, they had 5.3 years (1–14) of work at the district management unit (DMU) (see Table 2). The head-nurses coordinate all DMU’s activities such as the counselling, the opening of the HIV positive clients’ files, the presentation of the file to the doctor, the therapeutic patient education. They are also members of the hospital’s therapeutic committee and follow up people on antiretroviral treatment. The assistant-nurses are responsible of maintaining updated screening records and file keeping, and updating the medical records of patients on antiretroviral therapy. The lay counsellors are charge of the counselling (pre-test and post counselling), the therapeutic education, and the ART adherence supporting sessions.

**Table 2:** Demographic characteristics of research participants

Characteristics (*n* = 22)	*N* (%)
Participants per PVTCC	
PVTCC1	3 (14)
PVTCC2	4 (18)
PVTCC3	3 (14)
PVTCC4	4 (18)
PVTCC5	4 (18)
PVTCC6	4 (18)
Age in years; mean (SD)	42 (9)
Position	
Counselor	16 (73)
Nurse	6 (27)
Training	
Yes	21 (95)
No	1 (5)
Working experience in years; mean (SD)	5 (3)

1. Structure
  The structure of HTC services was analysed based on infrastructures, work environment, privacy and confidentiality during the counselling session, equipment, and training of the lay counsellors. The findings are presented in the same order.
  Infrastructures, work environment, privacy and confidentiality
    All the PVTCC are located within the district hospitals and are an integral component of the district management units. Only one PVTCC had enough and adequate rooms for the each lay counsellor to insure privacy and confidentiality during counselling sessions, this being a major concern among the participants: “*…First of all, our working environment is very stressful because I think in modern health facilities, things are organized so that the management of confidentiality is reasonably ensured. Many clients complain about the lack of privacy and confidentiality. The working environment must be less stressful. The client must feel comfortable and confident. So, when it comes into the counselling room, he must feel confident. Otherwise, it could affect his reaction later ». (Lay counsellor-PVTCC2)*
    In two PVTCCs, the lay counsellors share the same room and sometimes they receive their clients at the same time. Very often, in three PVTCCs, this office is cramped and not ventilated as mentioned by this lay counsellor: *« …firstly, the workplace is not adequate, then we work in precarious conditions and there is no ventilation here…» (Lay counsellor-PVTCC3).*
  Lack of equipment and materials
    None of the PVTCC had adequate equipment to perform rapid screening tests as recommended by the WHO. Nevertheless, one of the centers performed rapid screening test on-site. However, positive results must be confirmed by a second rapid screening test in the hospital lab. In the other centers, clients are directed to the DH lab for the rapid testing (blood sampling and testing) after the pre-test counselling. Almost in all of these centers, staffs experience a shortage in rapid test kits as mentioned by this lay counsellor: *« … there is another factor; it is the lack of reagents» (Lay counsellor-PVTCC1).* Furthermore, IEC material (i.e.,: video, poster) is obsolete or non-existent. One of the nurses commented: *« …If we have enough IEC materials, it suits us. Because there are patients who are illiterate, who do not speak French, who can neither read nor write and therefore do not understand what we say. With images, it should be better. That’s lacking. We had a VCR but it is no longer fashionable. If we had perhaps a more modern device to view, it would suit us » (Nurse-PVTCC5) ».*
  Training and qualification of lay counsellors
    Regarding human resources, head nurses under a physician’s supervision manage the PVTCCs. All the studied PVTCC have at least two counsellors as required. The lay counsellors are educated and come from various professional fields (education, sociology, economy, community health worker) and are generally trained by local NGOs. Almost all the counsellors are trained but there is no specific training curriculum accredited that leads to a formal registration as a PVTCC staff. The lay counsellors’ training differs both by length and content. Only selected counsellors trained by one NGO seem to have suitable training. Unfortunately, this NGO only trained only 10 counsellors in the city of Douala. Moreover, the continuing training of these personnel is not ensured. Even more serious, as reported by this lay counsellor, sometimes the staff is assigned to counselling without any initial training: *« …I still haven’t received a specific training. Gradually, as I see my colleagues work, I also adapt myself ». (Lay counsellor-PVTCC4)*
  Recognition and remuneration of lay counsellors
    Many of the lay counsellors who participated in this study complained about their working conditions and specifically their salary conditions. Apart from a few who are financially supported by the NGO, which trained them, the others continue to work without remuneration. From their point of view, Cameroon’s government promised to incorporate them into the civil service as reported by this lay counsellor: *« …The other big problem is the management of support staff as us. It is for love of the human being that we continue to work. But do we have to be sacrificed? We are a large group, we are everywhere in all hospitals. Some were incorporated. Others like me continue to volunteer» (Lay counsellor-PVTCC3) ».* They are considered as support staff in hospitals, without any recognition as explained by this lay counsellor: *«… It is the [name of NGO] program that makes us work in the hospital…the hospital does not take us into consideration. This aspect must be improved …» (Lay counsellor-PVTCC6).*
2. Process
  The findings related to the HTC process were related to the cost of the test, the consent to the test and, the waiting time for the test results.
  Cost of the screening test and consent
    Only one PVTCC clearly displays the testing fees. Moreover, the screening tests fees are not the same in all centers. Prices range from 500 FCFA, the official price to 2000 FCFA depending on the PVTCC as indicated by one of the participant: « … *When [the name of the NGO] was there, the test cost 500 FCFA. When they left, the hospital has just supported the cost of testing for a few months and then the meeting with the hospital’s staff, we tried to see the price that is affordable to all; and then we adopted the price of 2000 FCFA* …» *(Lay counsellor-PVTCC3).*
    Concerning the consent, according to few participants, it’s often happens that without being warned, the clients are sent to the counsellor’s office when they had just come for a medical consultation and not planned to undergo an HIV test. In the PVTTCs, only oral consent is requested. In none of them, written consent is sought. Moreover, in one PVTCC, even the oral consent is skipped because the clients are seen before in the pre-counselling session as explained by a lay counsellor: « *Elsewhere*, *when clients come for a screening test*, *at the reception desk, they are sent to the counsellor for an interview. After the interview, they are free to go or not to pay for the test. Here, though, clients begin with the payment before realizing that it was for the HIV test. Sometimes they did not want to do it but when they look at the money they have spent, they feel compelled » (Lay counselor-PVTCC1)*.
  Waiting time for results
    Despite the availability of rapid tests and their ease of use, none of the PVTCC delivers the results on the same day. The waiting time varies from 24 to 48 h for the screening test and several days or weeks for the confirmation in the case of a positive test. People who tested must therefore return to the PVTTC another day for the post-test counselling and results. This increases the waiting time, which discourages some clients. Therefore, they fail to return and abandon their results. According to most of the participants, this is partly explained by the workload at the hospital’s lab. Indeed, in addition to HIV tests the lab staff should perform all other hospital tests: This is why the blood samples are tested only later in the afternoon when the clients are gone and results available the next day at the best: « *I have asked the lab staff, they tell me they have too much work to do, they have not only the HIV screening tests. They take the blood samples in the morning and perform the tests later in the afternoon in order to make the results available the next day »* (*Lay counsellor-PVTCC5).*
    As this counsellor also said, sometimes clients have to return to the PVTCC not only one time but several times before getting their results: « *Here, the problem of results is the lab. Because someone did the test on Monday, for example, and he returns all week, the result is not available. Sometimes it takes a week or two weeks. That’s why the results are abandoned. Because logically, when someone did a test today, the day after, the result must be available… On the other hand, the biologist must validate the result is available…It’s the same for the confirmation test; the result can take two weeks. People return several times without being able to have their results » (Lay counsellor-PVTCC1).*

## Discussion

The WHO has identified five key elements to be respected and observed by all HTC services: consent, confidentiality, counselling, correct test results and connection (linkage to prevention, care and treatment) [7, 27]. For that purpose, a regular assessment of counselling and testing services is needed to ensure compliance with the standards recommended by the WHO [3, 7, 27]. This study aimed to identify factors that influence the quality of HIV counselling and testing services in district hospitals of the city of Douala in Cameroon using the Donabedian’s structure-process-outcome framework.

With the exception of one PVTCC with facilities that achieve the counselling in respect of privacy and confidentiality, the five other PVTCCs do not have adequate structures. Hence, the majority of participants in these PVTCCs have raised the issue of lack of privacy and confidentiality during counselling sessions. This is a health system concern, as the lack of privacy compromises confidentiality and contributes to making HIV counselling sessions less effective [8, 28]. This issue is particularly important when it concerns countries with stigmatization and discrimination against HIV positive persons [29, 30]. Indeed, in many communities, particularly in sub-Saharan Africa, stigmatization and discrimination may have consequences as devastating as the disease [29–31]. These findings had already been observed in previous studies which shown that people who perceive a lack of privacy are less likely to get tested know their HIV status and could also deter them from seeking care [28, 31–33]. Our results also show that in all PVTCCS, only verbal consent is required for the voluntary testing. However, in some situations, especially in the case of a provider-initiated testing, the client’s consent is not required. Most significantly, in one of PVTCC, the blood specimens of clients who are seeking voluntary testing are collected before the pre-test counselling and consent. “Informed consent” is defined as an individual’s right to make an informed, voluntary decision authorizing or refusing a medical intervention [34]. Informed consent has always been considered as the cornerstone that guarantees autonomy during the HIV testing process [35–37]. Despite the benefits of knowing one’s HIV status and the desire to make routine HIV testing for people visiting the health facilities of countries with a high HIV prevalence, as recommended by the WHO, the problem of informed consent for these people remains an ethical issue with various and varied positions [38–40]. Some authors argue that the changes are intended to devalue and undermine the principle of informed consent [41–44]. Proponents of opt-out testing think the classic way to obtain consent according to previously approved testing approaches might instead be a barrier [45–47]. Others argue that power inequalities between patients and health care providers can inhibit the ability of a patient to refuse HIV testing [35, 48].

In this study, most of the lay counsellors reported that they received adequate HIV counselling training. However, in the absence of a specific curriculum and national training standards, the counsellors’ training varied in duration and content, depending on the training organization and there is also a variation of training among lay counsellors in the same health facility. This situation calls into question the quality of the training and the counselling [7, 8].

Moreover, some lay counsellors work without training. Training is an important factor that allows lay counsellors be more confident and improve their performance [8, 9, 49]. Furthermore, our study also reveals a feeling of low recognition among the lay counsellors compounded by the lack of permanent positions and the absence or low pay. These results are similar to those of other studies in which the lay counsellors have also expressed dissatisfaction with their working conditions [8, 10, 50, 51].

In the HIV testing and counselling services, the lay counsellors help to reduce the workload of health workers and allow them to focus on other aspects of clinical care [7–9, 49]. The contribution of this specialized staff has increased HIV testing and counselling rates in many sub-Saharan Africa’s countries [7–9, 49]. However, the precarious working conditions and financial instability of this staff bring up the problem of the continuity and sustainability of this program. Maintaining a volunteer- based program may force the lay counsellors to choose between continuing to work and the economic necessity of finding additional paid employment elsewhere, a concern raised by Zachariah et al. [8, 49].

Finally, our study looked at the effect of the waiting time for results on the quality of counselling and testing services and more specifically on the return for the test results. Indeed, the majority of the participants recognized the waiting time for the results as a problem that leads people who get tested do not return for their results. Previous studies have reported an increasing rate in demand for testing, for the proportion of people who received post-test counselling and have been informed of their results, and that, following the introduction of rapid tests [52–58]. The results also showed that clients prefer attending the services where they can get their results without delay [57, 59–61]. This is consistent with the results of several studies reported that people who get tested preferred to receive their results on the test day [54, 59, 61].

This study has some limitations, which must be taken into account when interpreting the results. This study reported only the views and opinions of providers on the quality of HTC services. The perspectives of clients are not assessed. Participants’ experiences are self-reported; there could be a recall bias and a social desirability bias. The informants could be also influenced by the anxiety of the face-to-face interview and by the status of the interviewer (medical doctor). Despite these limitations, our study is the first to explore the quality of HIV testing and counselling in the Cameroonian context. A major strength of this study was the combination of two complemented data collection methods that enabled a triangulation of data, which enhanced the reliability and the validity of the findings. This study was conducted at the district hospitals of the city of Douala, the economic capital of Cameroon. Although these hospitals cover a large part of the population of the city, these are not the only places where it is possible to get tested for HIV. Thus the transferability of the study findings to other health facilities such as private, rural or upper-level health care facilities may be limited. Further researches carried out in these settings are needed to have an overview of the situation in the whole country. These researches should include both providers and clients.

## Conclusion

The findings of this study highlight some issues that hinder the quality of HTC services and should challenge the health authorities of Cameroon on the need to reorganize HTC services and the need to create a national HIV quality assurance program. These improvements imply to provide adequate infrastructure to ensure the privacy and confidentiality of counselling and that makes the work of lay counsellors less stressful. It would also be important to guarantee the client’s rights by requiring their consent before each test. Concerning the lay counsellors, having a national training curriculum, financial support and recognition should make them more confident and efficient in their work. Finally, avoiding the waiting time for the results after a screening or confirmatory test by making them available the same day should reduce the dropping of HIV test results and accelerate the initiation of treatment.

## Additional file

Additional file 1: Perceptions, opinions and experiences of HIV testing and counselling (HTC) providers regarding HTC services. Sociodemographic characteristics, job experience, job description, test, training, HIV testing and counselling procedures, organizational structure, return for HIV test results. (DOC 26 kb)
