# Supplementary material for: Challenges in the delivery of public HIV testing and counselling (HTC) in Douala, Cameroon: providers perspectives and implications on quality of HTC services
Source: BMC Int Health Hum Rights. 2017 Apr 8;17:9. doi: 10.1186/s12914-017-0118-2 (PMC5385024; doi:10.1186/s12914-017-0118-2)
Supplement: Additional file 1: — Perceptions, opinions and experiences of HIV testing and counselling (HTC) providers regarding HTC services. Sociodemographic characteristics, job experience, job description, test, training, HIV testing and counselling procedures, organizational structure, return for HIV test results. (DOC 26 kb) [file 12914_2017_118_MOESM1_ESM.doc]

Interview guide

1. Please, can you give me your age and your position in the prevention and voluntary testing and counselling center (PVTCC)? What is the highest level of education completed? How long have you been working in the PVTCC?
2. Describe your role in the organization?
3. Tell me about the training you received to do your job in HIV testing??
4. Describe the screening procedure (the different steps) for voluntary HIV testing in your organization.
5. At what step do you intervene in this procedure?
6. How do people who come for testing react to this procedure? (What are their reactions?) What do they tell you? How satisfied are they with this procedure? Explain.
7. What are the strengths of this procedure?
8. What are the points to be improved regarding this procedure?
9. How are voluntary testing results delivered in your organization?
   1. What do you know about the problem of returning for post-test counseling and test results after an HIV test in your organization?
   2. Why is it necessary to give a second appointment to people who get tested to see their test results in your organization?
10. How can we motivate people who are tested for HIV to return for their test results?
11. What do you think prevents them from coming back for their test results?
12. Do you have any other comments?

Thank you!!!
